# The Emerging Role of Nutraceuticals in Cardiovascular Calcification: Evidence from Preclinical and Clinical Studies

**DOI:** 10.3390/nu13082603

**Published:** 2021-07-28

**Authors:** Maristella Donato, Elisabetta Faggin, Francesco Cinetto, Carla Felice, Maria Giovanna Lupo, Nicola Ferri, Marcello Rattazzi

**Affiliations:** 1 Department of Pharmaceutical and Pharmacological Sciences, University of Padova, 35122 Padua, Italy; maristella.donato@studenti.unipd.it (M.D.); mariagiovanna.lupo@unipd.it (M.G.L.); nicola.ferri@unipd.it (N.F.); 2 Department of Medicine—DIMED, University of Padova, 35122 Padua, Italy; elisabetta.faggin@unipd.it (E.F.); francesco.cinetto@unipd.it (F.C.); carla.felice@aulss2.veneto.it (C.F.); 3 Medicina Generale I^, Ca’ Foncello Hospital, 31100 Treviso, Italy

**Keywords:** vascular calcification, nutrition, nutraceuticals

## Abstract

Cardiovascular calcification is the ectopic deposition of calcium-phosphate crystals within the arterial wall and the aortic valve leaflets. This pathological process leads to increased vascular stiffness, reduced arterial elasticity, and aortic valve stenosis, increasing the risk of cardiovascular diseases. Although cardiovascular calcification is an increasing health care burden, to date no medical therapies have been approved for treating or preventing it. Considering the current lack of therapeutic strategies and the increasing prevalence of cardiovascular calcification, the investigation of some nutraceuticals to prevent this pathological condition has become prevalent in recent years. Recent preclinical and clinical studies evaluated the potential anti-calcific role of nutraceuticals (including magnesium, zinc, iron, vitamin K, and phytate) in the progression of vascular calcification, providing evidence for their dietary supplementation, especially in high-risk populations. The present review summarizes the current knowledge and latest advances for nutraceuticals with the most relevant preclinical and clinical data, including magnesium, zinc, iron, vitamin K, and phytate. Their supplementation might be recommended as a cost-effective strategy to avoid nutritional deficiency and to prevent or treat cardiovascular calcification. However, the optimal dose of nutraceuticals has not been identified and large interventional trials are warranted to support their protective effects on cardiovascular disease.

## 1. Introduction

Cardiovascular (CV) calcification is defined as the ectopic deposition of calcium-phosphate crystals within the arterial tissues, mainly the aorta and its major branches and the aortic valve leaflets. Pathological calcification can occur in two distinct areas of the vessel wall: the intima (intimal calcification), which is inflammation-driven and typically associated with atherosclerotic plaques, or the media (arterial medial calcification) [1]. Medial calcification is prevalent in aging, diabetes mellitus, and chronic kidney disease (CKD) [2]. In particular, CKD is recognized as a public health problem worldwide, with a global estimated prevalence of 9–13% that is expected to further increase in the coming years [3,4]. In the aortic valve, calcification is the dominant process of advanced calcific aortic valve stenosis (CAVS), driving disease progression. CAVS is the most common heart valve disease in the Western world, with a prevalence of 12.4% in the elderly (over 75 years) [5]. Unfortunately, the disease becomes symptomatic only at the severe stage when it requires the substitution of the valve. The prevalence of severe CAVS increases with advancing age, from 0.02–0.1% in patients aged 18–44 years to 2.8–4.6% in the elderly (over 75 years) [6]. Compared to the general population, CKD patients are at a higher risk of developing CAVS, which is associated with lower survival. Considering the higher life expectancy and the rapid aging of the population, the prevalence and impact on public health of valvular calcification are expected to further increase in the near future [7,8,9].

Arterial medial calcification increases the stiffness and reduces the elasticity of the vessels, and it has been associated with an increased risk of CV events and mortality, not only in high-risk subjects but also in the general population [10,11]. This association is particularly evident in CKD patients, where vascular calcification has an overall prevalence of 60% and significantly affects the risk of CV events and all-cause mortality [12]. Moreover, in this high-risk population, the progression of vascular calcification is faster with worsening CKD [13]. 

On these bases, it appears that interventions preventing or limiting CV calcification might have a relevant impact on CV disease protection, especially in a high-risk population.

## 2. Purpose

The development of effective treatments for CV calcification is an urgent clinical need, considering the current lack of medical therapies and the increasing prevalence of the disease. In recent years, preclinical and clinical studies have been performed to evaluate the potential beneficial effects of nutraceuticals supplementation on the prevention and treatment of vascular calcification. The present review briefly summarizes the main risk factors and pathophysiological mechanisms for the development of CV calcification and the current knowledge in the clinical management of this pathological condition. In the main part, emphasis is placed on the nutraceuticals with the most relevant preclinical and clinical data, including magnesium, zinc, iron, vitamin K, and phytate.

## 3. Risk Factors for Cardiovascular Calcification

Vascular calcification was initially thought to be only an age-related passive process, caused by the deposition of hydroxyapatite (HA) crystals in the extracellular matrix (ECM). Although the pathogenesis of ectopic calcification in the medial layer of the vessels and in the aortic valve is still unclear, increasing evidence supports the hypothesis that it is also an active and cell-mediated process similar to skeletal bone formation.

In the medial layer of the vessels, vascular smooth muscle cells (VSMCs) can undergo differentiation into an osteochondrogenic phenotype, characterized by the downregulation of smooth muscle contractile proteins, and the expression of osteogenic markers (such as ALP, Runx2, BMPs). The transdifferentiation of VSMCs can be promoted by several different factors, including oxidative stress, an imbalance between calcification promoters and inhibitors, cellular senescence, and hyperphosphatemia [14]. Once VSMCs are differentiated, they secrete calcifying extracellular vesicles rich in calcium-phosphate crystals, promoting further mineralization in the ECM [15]. In addition, osteoblast-like VSMCs can release apoptotic bodies, which act as scaffolds for ECM mineralization [16].

In the aortic valve, increased mechanical stress and reduced shear stress can induce endothelial dysfunction, which favors the infiltration of monocytes, immune cells, and lipoproteins. Therefore, the first phase of CAVS is characterized by oxidative stress, inflammatory response, and lipid deposition in the sub-endothelium and in the fibrosa layer of the tissue [17]. In the second phase, fibrosis and calcification assume a prominent role in the progression of the disease. Valvular calcification is driven by the osteogenic differentiation of VICs, promoted by several signaling pathways (including Wnt/β-catenin, RANK/RANKL, and BMPs) [18,19]. The osteogenic phenotype of VICs is similar to osteochondrogenic VSMCs, characterized by an increased expression of osteogenic markers.

Traditional risk factors for CV calcification are dyslipidemia, inflammation, diabetes, obesity, and hypertension [20]. In addition to this, ectopic calcification is also associated with several non-traditional risk factors in the CKD population [21]. In fact, in CKD patients, a dysregulated mineral metabolism, the loss of calcification inhibitors (including MGP, fetuin-A, pyrophosphate), and the activation of inflammatory pathways contribute to the development and progression of vascular calcification [22]. Moreover, the renal function impairment and the consequently reduced excretion of phosphate lead to hyperphosphatemia, a condition that favors mineral deposition in both vascular walls and the aortic valve and the phenotypic switch of VSMCs [23]. Phosphate absorption and excretion also depend on a network involving calcium, vitamin D, and parathyroid hormone (PTH), factors that are dysregulated in pathological conditions like CKD [22].

## 4. Pharmacological Approaches for the Treatment of Vascular Calcification

Concerning valvular calcification, to date, no effective pharmacological treatments have proven to halt or delay it and aortic valve replacement represents the only option to improve clinical outcomes [24].

The pathophysiological mechanisms leading to vascular calcification are still unclear, therefore, at present, there is no comprehensive treatment to prevent or counteract it. The current strategies are based on the modulation of its key regulators, such as vitamin D, calcium, phosphate, through calcimimetics, vitamin D analogs, and phosphate binders [21,25]. Phosphate binders limit dietary phosphate absorption, lowering its serum levels, and are used in clinical practice to manage hyperphosphatemia in CKD patients. It has been demonstrated that excessive oral intake of calcium, also by calcium-containing phosphate binders, increases the risk for vascular calcification; thus, calcium-free phosphate binders should be preferred [26,27]. However, it is uncertain whether phosphate binders can prevent ectopic calcification and reduce the risk of CV events, as underlined by a recent meta-analysis [28]. A recent review suggests that calcium-free phosphate binders and low-dose vitamin D analogs combined with calcimimetics may reduce the severity of vascular calcification [29], but clinical evidence is still insufficient to recommend this strategy.

Therefore, based on current evidence, optimal treatments for vascular calcification remain to be found. A detailed description of phosphate binders, calcimimetics, and vitamin D analogs has been given by other recent reviews [30,31,32] and goes beyond the purpose of the present paper.

In recent years, emphasis has been placed on the role of some nutraceuticals to improve health and to prevent diseases. It is well established that adequate serum levels of nutraceuticals maintain CV health. Their overall levels are dependent on oral intake, gut absorption, tissue pools, renal excretion, and several other factors, strictly correlated to the nutritional and medical status of the subject. Considering that nutritional deficiency is common in high-risk populations and that preclinical evidence has detected anti-calcific effects of some nutraceuticals (such as magnesium, zinc, iron, vitamin K, and phytate), several preclinical and clinical studies have been performed to assess the potential effects of their supplementation for vascular calcification.

## 5. Nutraceuticals for Cardiovascular Calcification

### 5.1. Magnesium

Magnesium, one of the most abundant cations in the human body (25 g), is widely distributed in plant foods, such as green leafy vegetables, legumes, nuts, seeds, and whole grains [33]. Magnesium is an essential mineral for many physiological functions. Mainly found in the ionized form, it acts as a counter ion for ATP and as a cofactor in several enzymatic reactions required for the structural function of nucleic acids, proteins, and mitochondria. Moreover, magnesium contributes to muscle contraction/relaxation, the release of neurotransmitters, regulation of vascular tone, and bone formation [34].

The role of magnesium in CKD-associated vascular calcification has been assessed in several preclinical studies. An ex vivo/in vitro study on both VSMCs and aortic segments determined the inhibitory action of magnesium on phosphate-mediated vascular calcification [35].

**Figure 1:**
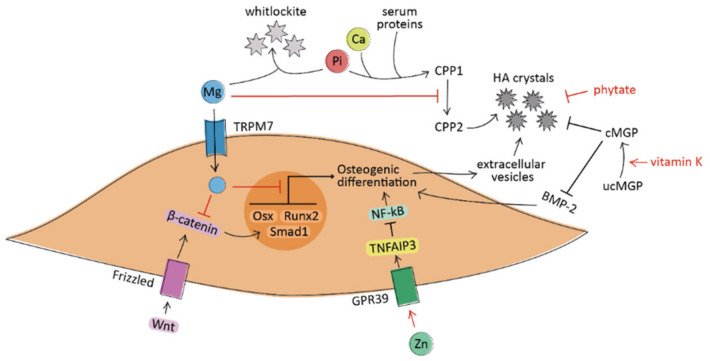
A simplified schematic representation of the processes under investigation leading to the osteogenic differentiation of VSMCs in vascular calcification. Magnesium (Mg) acts at the extracellular level by preventing the maturation of calciprotein particles (CPPs) and by forming whitlockite crystals with phosphate ions (Pi). At the intracellular level, Mg decreases the expression of osteogenic genes (such as Osterix, Smad1, and Runx2) and inhibits pro-calcific pathways (including Wnt/β-catenin). Zinc (Zn) induces the suppression of NF-κB through the activation of the GPR39/TNFAIP3 signaling pathway. Vitamin K promotes the carboxylation and activation of the matrix Gla protein (MGP), which avoids the formation of hydroxyapatite (HA) crystals and prevents the pro-calcific action of BMP-2. Phytate acts as crystallization inhibitor by binding to HA crystals. The regular arrow denote “activation”, and the T-shaped arrow stands for “inhibition”.

The molecular mechanisms by which magnesium counteracts vascular calcification are still not completely understood, but they seem related both to intracellular and extracellular levels of this micronutrient. The active role of intracellular magnesium as an anti-calcific agent is supported by in vitro evidence. It has been shown that magnesium prevents VSMCs transdifferentiation by decreasing the expression of pro-calcification markers (including Osterix, Smad1, and Wnt/β-catenin pathway) [36,37], by increasing calcification inhibitors (including MGP and osteoprotegerin), and by improving VSMCs viability [38,39]. These effects are mediated by the entry of magnesium into the cells via transient receptor potential melastatin-7 (TRPM7), whose activity is reduced in osteogenic VSMCs and restored in the presence of magnesium [40]. In addition, magnesium can act as an anti-crystallization agent at the extracellular level; it avoids the formation of hydroxyapatite from amorphous calcium phosphate, blocking the process of crystal nucleation [41]. Firstly, magnesium is a natural calcium antagonist; thus, it can substitute calcium ions in hydroxyapatite, favoring the formation of whitlockite, less pathogenic crystals [42]. In addition, magnesium avoids the transition of amorphous Ca–Pi particles into crystalline hydroxyapatite. In particular, this action is due to the inhibition of calciprotein particles (CPPs) maturation from amorphous CPP1 to crystalline CPP2 [43] (Figure 1).

It has been recommended to consume at least 400–420 mg/day of magnesium for men and 310–320 mg/day for women [33]. The regular consumption of magnesium is fundamental to maintain the physiological processes and to prevent magnesium deficiency. In addition, recommended values of serum magnesium levels have been considered relevant for its protective effects on CV health. Hypomagnesemia is indeed associated with an increased incidence of hypertension, type 2 diabetes, and metabolic syndrome in the general population [44]. Moreover, dietary magnesium intake is inversely correlated with the risk of CV events and all-cause mortality [45,46]. A significant relationship between low serum magnesium levels and all-cause mortality has also been found by a recent meta-analysis of 20 clinical trials involving CKD and dialysis patients [47].

The relationship between magnesium intake and vascular calcification was investigated in the Framingham Heart Study, which found a strong inverse association [48]. This study, together with the above-described preclinical findings, provided evidence for performing subsequent trials to assess the effects of magnesium supplementation on vascular calcification, especially in high-risk patients. Some clinical trials investigated the potential effects of long-term magnesium supplementation (more than 6 months) on the progression of atherosclerosis in hemodialysis patients; the trials measured carotid intima-media thickness (IMT), a well-established marker of subclinical atherosclerosis, finding a significant improvement in this parameter after magnesium consumption [49,50]. Therefore, magnesium is likely to play a protective role in the development of atherosclerotic lesions. However, the studies were limited by small sample size (44 and 54 patients respectively); thus, these preliminary findings should be confirmed by larger-scale trials.

Magnesium is used in clinical practice as a phosphate binder for its ability to bind dietary phosphate in the intestinal tract. In few pilot studies, oral magnesium carbonate was administered to hemodialysis patients as a magnesium-containing phosphate binder, providing positive outcomes on vascular calcification [51,52] (Table 1). The protective effect of magnesium, however, seems not to be limited to its phosphate-binding capacity; a recent in vivo study on a uremic mouse model observed a decrease in vascular calcification after intraperitoneal magnesium administration, suggesting a direct effect of magnesium independently on its intestinal action [53]. Taken together, these findings provided a strong rationale for investigating in further clinical trials whether dietary magnesium could reduce the risk of vascular calcification and CV events in CKD patients. Recently, a randomized controlled trial on predialysis patients observed that the long-term supplementation of magnesium oxide could effectively slow the progression of coronary artery calcification [54]. These data represent the strongest evidence so far of the anti-calcific action of magnesium. Currently, the effects of magnesium on the progression of coronary artery calcification in predialysis subjects are also being assessed in the MAGiCal-CKD randomized trial [55] (Table 2).

Based on current evidence, magnesium supplementation seems a promising strategy for the management of vascular calcification. In addition, it would be affordable and easily accessible to patients due to the cost-effectiveness and the wide availability of this mineral. However, larger randomized clinical trials are warranted before suggesting its supplementation to CKD patients.

Of note, an adequate magnesium intake is also necessary for bone health: 60% of total magnesium is stored in the bone, where it maintains proper mineral density. However, there is increasing evidence supporting the hypothesis that magnesium supplementation in high doses may counteract HA crystals formation even in bone, leading to mineral defects such as osteomalacia [62,63]. The tolerable upper intake levels for supplemental magnesium are 350 mg/day [33], but further studies are needed to investigate whether an increase in dietary magnesium may decrease bone mineral density as a side effect.

Moreover, it is important to underline that dietary intake of magnesium is not always correlated with its serum concentration and even serum levels of magnesium do not reflect its total body content. Magnesium homeostasis depends on the balance between gut absorption, renal excretion, and tissue pools (mainly in bone), which may vary from one subject to another according to the patient’s medical condition [34]. In patients with CKD, the loss of renal function is counteracted by compensatory mechanisms, which maintain the serum levels of magnesium in the normal range or may even cause hypermagnesemia when renal excretion is further decreased [64]. On the other hand, some drugs commonly used by CKD patients can decrease gastrointestinal absorption of magnesium, potentially contributing to hypomagnesemia [65]. Therefore, several factors (such as the medical status, CKD stage, and drug administration) should be considered when an oral supplementation of magnesium is suggested to CKD patients.

As suggested by Hamano and colleagues [66], further research should focus on the clinical impact of magnesium supplementation on CV events and mortality. Moreover, the optimal dose of dietary magnesium should be determined, in order to maximize the benefits without causing potential side effects. More recently, new oral formulations of magnesium have been developed in order to improve its bioavailability and to ameliorate its physiological inhibitory effect on vascular calcification [67].

### 5.2. Zinc

Zinc, one of the most abundant divalent cations in the body (2–4 g), can be found in a variety of foods, including oysters, red meat, seafood, beans, and dairy products. The recommended oral consumption of zinc is 8 mg/day for women, 11 mg/day for men, and the tolerable upper intake level for adults is 40 mg/day [68]. Zinc is an essential micronutrient for several physiological functions, including the catalytic activation of zinc-containing metalloenzymes, the regulation of cellular homeostasis and intracellular signaling, the preservation of the active conformation of DNA-binding proteins, and transcription factors. Moreover, it exerts strong antioxidant and anti-inflammatory effects [69].

**Figure 2:**
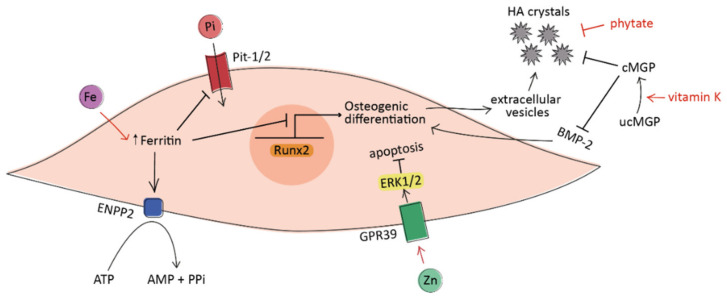
A simplified schematic representation of the processes under investigation leading to the osteogenic differentiation of VICs in vascular calcification. Iron (Fe) acts at the intracellular level via ferritin, which prevents phosphate uptake and inhibits VICs osteogenic differentiation. Moreover, ferritin promotes the production of anti-calcific pyrophosphate (PPi) from ATP by activating the enzyme ENPP2. Zinc (Zn) induces VICs apoptosis through the activation of the GPR39-ERK1/2 signaling pathway. Vitamin K promotes the carboxylation and activation of matrix Gla protein (MGP), which avoids the formation of hydroxyapatite (HA) crystals and prevents the pro-calcific action of BMP-2. Phytate acts as crystallization inhibitor by binding to HA crystals. The regular arrow denotes “activation”, and the T-shaped arrow stands for “inhibition”.

The anti-calcific effect of zinc is supported by in vitro evidence on both VSMCs [70] and VICs [71]. It seems likely that zinc does not directly inhibit calcium phosphate precipitation but acts indirectly through the activation of cellular mechanisms. In particular, the anti-calcific effect in VSMCs depends on the GPR39-mediated upregulation of TNFAIP3 protein levels and the subsequent suppression of NF-κB, a pro-inflammatory transcription factor involved in their osteochondrogenic differentiation [72] (Figure 1). In cultured VICs, zinc supplementation reduces the calcification process through the inhibition of apoptosis and the activation of the GPR39-dependent ERK1/2 signaling pathway [71] (Figure 2).

In preclinical studies, reduced dietary zinc intake promoted vascular inflammation and arterial plaque formation [73], while zinc supplementation exerted an anti-atherosclerotic role [74]. Moreover, a long-term supplementation of zinc was effective in reducing vascular calcification in hyperphosphatemic mice [72].

Taken together, these experimental studies provide evidence on the role of adequate serum zinc levels for maintaining CV health. Unfortunately, zinc deficiency is a common condition, especially in developing countries. Zinc insufficiency is caused by low dietary zinc intake, decreased absorption, or increased loss of zinc, and may be associated with the development of diabetes, cancer, gastrointestinal disease, and CKD [75]. More recently, zinc deficiency has also been correlated to hypercholesterolemia, high blood pressure, and inflammation, leading to an increased risk of CV diseases. In CKD patients, the decrease in the circulating levels of zinc is probably due to a higher urinary excretion; moreover, the reduction in baseline plasma concentration has been recently associated with a higher probability of renal function decline [76].

Based on this evidence, increasing zinc intake might be proposed to avoid the detrimental effects of its deficiency, especially in high-risk subjects. However, it should be taken into account that serum levels of zinc are poorly correlated to its oral intake. For example, athletes have lower serum zinc concentration, despite the higher total dietary zinc intake [77]; a weak relationship between dietary consumption and serum levels of zinc has also been found in a study involving 408 healthy girls [78]. These findings are probably due to the differences in bioavailability from dietary sources and the different metabolism of the subjects. Therefore, these aspects should be considered before suggesting an oral supplementation of zinc.

The relationship between oral zinc intake and arterial calcification in humans was investigated in few clinical studies. A recent cross-sectional study associated a higher dietary consumption of zinc with a lower probability of having severe abdominal aortic calcification [79]. Moreover, an inverse correlation was observed between circulating levels of zinc and serum calcification propensity in patients with type 2 diabetes [80]. Increasing zinc consumption might be a simple but effective approach to reduce the progression of vascular calcification and the risk of CV diseases. Despite the positive findings, there is currently insufficient evidence to recommend its supplementation for preventing CV diseases, as reported by a recent review [81]. Further clinical studies are required to confirm the association between hypozincemia and vascular calcification and the potential benefits of zinc supplementation.

### 5.3. Iron

Iron is an essential component of hemoglobin and is necessary for several physiological processes, including DNA synthesis, enzymatic activities, and mitochondrial energy production. Dietary iron is available in two forms: heme, derived from meat and fish, and nonheme, obtained from cereals, legumes, fruits, and vegetables [82]. The recommended daily intake is 8 mg for men and reaches 18 mg in women up to 50 years; the tolerable upper intake level from food and supplements for adults is 45 mg/day [83].

Increasing preclinical evidence has investigated the effects of iron on CV calcification. In vitro, iron prevented phosphate-induced osteoblastic differentiation in both VSMCs and VICs via ferritin and its ferroxidase activity [84,85]. In the aortic valve, in particular, ferritin effectively enhanced pyrophosphate production and reduced cellular phosphate uptake [85] (Figure 2). Pyrophosphate is a well-established calcification inhibitor, avoiding the growth of HA crystals [86]. The anti-calcific effect of iron was also confirmed by an in vivo study, where parenteral iron administration inhibited the development of vascular calcification in uremic rats [87].

Maintaining adequate serum values of iron is fundamental for several physiological processes, considering also that iron might play a protective role for vascular calcification. On the other hand, inadequate iron bioavailability, causing an iron deficiency, has severe clinical consequences. Iron deficiency is common in CKD patients and leads to anemia; for this reason, the supplementation of iron is currently prescribed to these subjects. While iron is exclusively administered intravenously in dialysis patients, in non-dialysis subjects the route of administration is still controversial. Oral supplementation is inexpensive and easier to administer but is frequently associated with gastrointestinal adverse events that can limit both the efficacy and the adherence to therapy [88]. Therefore, despite the disadvantages of a parenteral administration, a recent systematic review suggests the use of intravenous iron as standard care for treating CKD-associated anemia even in non-dialysis-dependent CKD patients, stages 3 to 5 [89]. 

Recently, two oral preparations (iron citrate and sucroferric oxyhydroxide) have become available as effective iron-based phosphate binders, approved for treating hyperphosphatemia in CKD patients [90]. This new class of phosphate binders has the advantages of being calcium-free, avoiding the side effect of hypercalcemia, and of enhancing iron availability, also improving anemia [91]. In addition to these properties, increasing preclinical evidence suggests a beneficial effect of these phosphate binders also for vascular calcification. Recent in vitro studies by Ciceri and colleagues analyzed the direct effect of iron citrate, independently from its phosphate chelating action, on phosphate-induced calcification of VSMCs. Iron citrate prevented and partially reverted the high phosphate-induced osteochondrogenic shift of the ECM [92]. Moreover, the exposure of VSMCs to iron citrate inhibited calcium deposition through the prevention of apoptosis and the induction of autophagy [93]. Of note, iron was able not only to prevent calcium deposition but also to avoid further progression when calcification was already established [94]. Based on these findings, it seems likely that iron can act on vascular calcification independently of the grade of VSMCs differentiation.

The anti-calcific properties of sucroferric oxyhydroxide and iron citrate were further confirmed by in vivo studies, which demonstrated their effectiveness in preventing the progression of ectopic calcification in uremic rats [95,96].

These preliminary findings suggest that iron administration in patients with hyperphosphatemia and iron deficiency might also prevent vascular calcification in this high-risk population. However, this hypothesis should be assessed in further preclinical studies and clinical trials, which are currently lacking.

Furthermore, the optimal dose of iron for supplementation should be determined to avoid an iron overload, which has negative effects on bone metabolism. In fact, iron can decrease bone mineralization by inhibiting osteoblast differentiation, eventually leading to osteopenia and osteoporosis. This inhibitory action depends on iron-induced upregulation of ferritin and its ferroxidase activity, similarly to what happens in VSMCs [97,98]. Moreover, excess iron enhances oxidative stress, which in turn may promote vascular calcification [98].

Thus, the supplementation of iron for preventing or halting vascular calcification to date is not supported by clinical studies and requires further evidence before being suggested, although the preclinical findings are promising. Moreover, the optimal dose to avoid side effects remains to be found.

### 5.4. Vitamin K

Vitamin K refers to a group of liposoluble vitamins: vitamin K1 (phylloquinone) naturally found in fruits and vegetables, and vitamin K2 (menaquinones) produced by gut bacteria [99]. The adequate intake of vitamin K is considered 120 µg/day for men and 90 µg/day for women [100].

Vitamin K is a required cofactor for the γ-carboxylation (and thereby activation) of several vitamin K-dependent proteins, among them the matrix Gla protein (MGP), a potent inhibitor of soft-tissue calcification. The activated carboxylated form of MGP (cMGP) binds to calcium ions and interacts with bone morphogenetic protein-2 (BMP-2), avoiding, respectively, the formation of HA crystals and the osteogenic differentiation of cells [101,102] (Figure 1 and Figure 2). There is experimental evidence of the role of cMGP in preventing vascular calcification; in fact, the silencing of the MGP gene promoted the osteoblastic differentiation of VICs [103] and the development of arterial calcification [104].

In addition to the carboxylation and activation of MGP, vitamin K exerts anti-inflammatory and lipid-lowering properties, which may also contribute to its potential beneficial effects. In particular, the serum levels of vitamin K1 have been inversely correlated with circulating inflammation markers, such as ICAM-1, C-reactive protein, and IL-6 [105]. Moreover, vitamin K2 has been associated with decreased cholesterol biosynthesis and increased low-density lipoprotein receptor (LDLR) [106].

A low intake of vitamin K or the consumption of vitamin K antagonists (such as warfarin) can lead to vitamin K deficiency, which reduces the available amount of cMGP [107,108]. In patients undergoing hemodialysis, reduced serum levels of cMGP have been associated with an increased risk for vascular calcification [109]. Vitamin K deficiency is a common condition in CKD patients, where it is significantly associated with vascular calcification [110]. In this high-risk population, the insufficiency of vitamin K is often the result of dietary restrictions or the frequent use of warfarin as an anticoagulant [99]. Considering also that vitamin K antagonists promote CV calcification and aortic valve (AV) degeneration [111,112,113], they should be substituted with novel oral anticoagulants (NOACs), which are preferable even for maintaining bone health [114].

Thus, the dietary supplementation of vitamin K might be proposed to avoid vitamin K deficiency and to prevent ectopic calcification, especially in high-risk subjects. In this case, the upper limit intake is 200 µg/day [115]. The effects of oral vitamin K supplementation have been assessed in randomized controlled trials with conflicting results. Vitamin K2 administration failed to decrease the progression of vascular calcification in patients with advanced CKD [56] or diabetes [57], but these studies were limited by short-term follow-up and a small sample size. In a long-term trial, daily supplementation of vitamin K1 for 3 years showed positive outcomes in elderly people with pre-existing vascular calcification [58]. The administration of vitamin K1 was also associated with slower progression of AV calcification in a small size study [59]. The clinical studies are described in detail in Table 1. Despite these positive preliminary findings, a recent systematic review has underlined the lack of sufficient evidence to support a beneficial effect of vitamin K in preventing the progression of calcification and atherosclerosis [116]. Moreover, in a very recent randomized study (K4Kidneys trial) involving patients with advanced CKD, vitamin K2 failed to improve vascular stiffness, a marker of vascular health [117].

Clinical trials are currently ongoing to evaluate the potential efficacy of vitamin K in preventing vascular calcification in hemodialysis patients (iPACK-HD, TReVasc-HDK) [118,119] and in subjects with coronary heart disease (VitaK-CAC) [120]. Moreover, the AVADEC and BASIK2 randomized trials are investigating the effects of vitamin K2 on the progression of AV calcification [121,122]. These trials are described in detail in Table 2. If the trials give positive outcomes, they will provide an effective preventive treatment for ectopic calcification a simple, safe, and readily available compound that can be easily supplemented in patients. Evidence of successful regression of ectopic calcification will support larger and longer-term trials.

### 5.5. Phytate

Phytate (InsP6), the hexasodium salt of myo-inositol hexaphosphate, is a naturally occurring compound present in unprocessed plant foods, such as whole grains, legumes, seeds, and nuts [123]. 

Phytate can chelate calcium ions with high affinity, a property that is beneficial for ectopic calcification; in fact, it binds in the growing site of HA crystals blocking their additional growth without reducing the circulating calcium levels [124] (Figure 1 and Figure 2). The structural similarities of phytate with other polyphosphates (such as bisphosphonates and pyrophosphate) give it similar anti-calcific activity, but with the highest potency as a crystallization inhibitor [125]. The different inhibitory activity may be explained by the higher number of phosphates in the chemical structure, increasing the possibility to bind calcium ions [126].

The anti-calcific effects of phytate on ectopic calcification were assessed in preclinical and clinical studies. In rats, oral phytate significantly reduced calcium deposition in the aorta and heart tissues [127,128,129], while its absence in the diet caused renal calcification [130]. A prospective cross-sectional study evaluated the relationship between physiological levels of phytate and CV calcification, concluding that an adequate consumption of phytate could prevent abdominal aortic calcification in CKD patients [131].

It has been observed that the levels of phytate in tissues and biological fluids mainly depend on the dietary intake rather than on the endogenous synthesis [132,133]. In fact, the overall phytate levels are limited by its reduced absorption in the gastrointestinal tract, considering that at physiological pH the compound is strongly negatively charged and that most oral phytate is degraded by plant food phytases during digestion [123]. 

Therefore, clinical trials evaluated the possibility to supplement phytate for maintaining adequate levels and for preventing or targeting ectopic calcification, especially in high-risk patients. A prospective randomized trial (CALCIFICA, NCT01000233) assessed the effects of an oral supplementation of phytate on patients with CAVS, but the results of the study are still unknown [134].

Of note, phytate is considered an anti-nutrient for its tendency to strongly bind to polyvalent cations such as calcium, magnesium, zinc, and iron, reducing their intestinal absorption and bioavailability. This characteristic is not damaging in the context of a balanced diet, but it can become detrimental in plant-based diets with a high intake of phytate [135,136]. Therefore, before suggesting an oral supplementation of phytate, the patient’s status and diet should be considered. Furthermore, the concomitant supplementation of phytate and cationic micronutrients should be avoided.

Although the dietary consumption of phytate might be recommended for mild forms of calcification, it could be inadequate for the prevention of severe ectopic calcification, considering its poor oral bioavailability. A parenteral administration is required for reaching supra-physiological phytate concentrations [137]; thus, an intravenous formulation of phytate (SNF472) has been developed for the treatment of vascular calcification and calciphylaxis in patients undergoing hemodialysis. SNF472 has been shown to reduce the progression of ongoing calcification in a dose-dependent manner in cultured VICs [138] and VSMCs [124]. Infusions of SNF472 can reduce calcification in the aorta and heart by at least 60% both in vitamin D-treated rats [139] and uremic rats [140]. Recently, randomized trials on healthy volunteers and dialysis patients have assessed the safety, tolerability, and efficacy of SNF472 in slowing the deposition of calcium in the heart and coronary arteries [60,61] (Table 1). Phytate may provide a novel strategy to target ongoing ectopic calcification in a selective and effective way and could be administered to subjects with CKD during hemodialysis, but more clinical research is needed to confirm this hypothesis.

## 6. Conclusions and Future Perspectives

Cardiovascular calcification represents an increasing health care burden, but to date, specific medical therapies for treating or preventing it have yet to be found. The pharmaceutical treatments currently administrated in clinical practice (such as vitamin D analogs, phosphate binders, and calcimimetics) have inadequate clinical evidence to recommend their use for vascular calcification.

The protective role on CV health of an adequate intake and serum values of some nutraceuticals in the general population is well established. Considering the current lack of therapeutic strategies and the increasing prevalence of CV calcification, the investigation of some nutraceuticals to prevent this pathological condition is becoming prevalent in recent years. Increasing clinical trials, reported in the present review, have found an inverse correlation between dietary intake or serum levels of some nutraceuticals (such as magnesium, zinc, vitamin K) and the progression of vascular calcification. These studies provide further evidence that maintaining the correct values of micronutrients might be a valuable strategy for the prevention of CV diseases. This aspect is particularly relevant in high-risk populations, such as CKD patients, which are frequently characterized by a nutritional deficiency of several micronutrients.

Increasing preclinical evidence, reported in the present review, has found significant inhibitory effects of magnesium, zinc, iron, vitamin K, and phytate on the development and progression of CV calcification. These findings, together with the frequent deficiency of these nutraceuticals in CKD subjects, support the hypothesis of an oral supplementation firstly to avoid the detrimental effects of a nutritional insufficiency and secondary as a cost-effective strategy to prevent or treat ectopic calcification, especially in this high-risk population.

Concerning the supplementation of iron and zinc, interventional controlled trials are lacking and should be performed to confirm the promising preclinical findings. The effects of a supplementation of magnesium and phytate on ectopic calcification have been assessed in clinical trials with preliminary positive results. In regard to vitamin K, several interventional studies have been performed with conflicting findings, summarized in Table 1. Thus, the current clinical evidence is still insufficient to suggest the supplementation of nutraceuticals for preventing or slowing the progression of vascular calcification, especially in high-risk subjects. Some interventional trials are currently ongoing to evaluate the effects of nutraceuticals supplementation (summarized in Table 2); if successful, they will provide further evidence for performing larger and longer-term trials.

It is important to underline that the nutraceuticals reported in the present review might be used as preventive strategies for high-risk populations rather than therapeutic agents. Among the above-reported nutraceuticals, phytate and iron seem to be effective also in slowing the further progression of the disease when calcification is already established; they might also be used as therapeutic agents, but further evidence should confirm this hypothesis. Of note, to date trials addressed to the general population are missing and might be done to evaluate whether supplementation of these nutraceuticals is beneficial even for them.

Another important aspect to consider is the evaluation of the optimal dose of the nutraceuticals for preventing or targeting CV calcification without the side effects caused by an over-dosage. In fact, an iron or magnesium overload can interfere with physiological processes, including bone mineralization. Moreover, the serum levels of the nutraceuticals are only partially dependent on the oral intake, while they are correlated to several other factors, including tissue pools, gut absorption, renal excretion, and co-administration of drugs. Therefore, an evaluation of the patient’s nutritional and medical status should be conducted before suggesting the supplementation of nutraceuticals.

Thus, to date, an optimal dietary supplementation of nutraceuticals for preventing vascular calcification has not been identified and further interventional studies may help to find a solution for this relevant pathological condition.

## Figures and Tables

**Table 1:** Interventional trials performed to evaluate the effects of nutraceuticals on vascular calcification.

Reference	Intervention	Phase	Population	Enrolment	Findings
[51]	Magnesium carbonate plus calcium acetate vs. calcium acetate alone	pilot	Hemodialysis patients	72	Magnesium retards the progression of arterial calcification
[52]	Magnesium carbonate/calcium carbonate	pilot	Hemodialysis patients, CAC scores >30	7	Magnesium may have a favorable effect on the progression of CAC
[54]	Magnesium oxide vs. placebo	-	CKD patients with risk factors for CAC	125	Magnesium oxide is effective in slowing CAC progression
[56]	Vitamin K2 plus vitamin D vs. vitamin D	IV	Non-dialyzed CKD patients	42	Vitamin K2 does not affect the progression of calcification
[57]	Menaquinone-7 vs. placebo	-	Type II diabetes and CVD	68	Menaquinone-7 does not decrease vascular calcification
[58]	Vitamin K1 vs. placebo	III	Healthy subjects (>60 years) with CAC	452	Vitamin K1 slows the progression of CAC
[59]	Vitamin K1 vs. placebo	III	Asymptomatic AVC	99	Vitamin K1 might decelerate the progression of AVC
[60]	iv SNF472 vs. placebo	I	Healthy volunteers and dialysis patients	28	SNF472 administration reduces HA crystallization potential
[61]	iv SNF472 vs. placebo	IIb	Hemodialysis patients	274	SNF472 attenuates CAC progression and AVC

iv, intravenous; CKD, chronic kidney disease; CAC, Coronary Artery Calcification; AVC, aortic valve calcification; CVD, cardiovascular disease; HA, hydroxyapatite.

**Table 2:** Ongoing clinical trials evaluating the effects of nutraceuticals on vascular calcification.

Trial Name	NCT	Intervention	Phase	Population	Enrolment	Primary Outcome
MAGiCal-CKD	NCT02542319	Magnesium hydroxide vs. placebo	II-III	Predialysis CKD	250	Change in CAC score at 12 months
iPACK-HD	NCT01528800	Vitamin K1 vs. placebo	II	Hemodialysis patients	85	Progression of CAC at 12 months
TReVasc-HDK	NCT02870829	Menaquinone-7 vs. placebo	II	Hemodialysis patients	178	Difference in CAC score at 18 months
VitaK-CAC	NCT01002157	Menaquinone-7 vs. placebo	-	CAC score between 50 and 400	180	Change in CAC score at 12 and 24 months
AVADEC	NCT03243890	Menaquinone-7 vs. placebo	-	AVC score above 300, without AVS	389	Progression of AVC at 2 years
BASIK2	NCT02917525	Vitamin K2 vs. placebo	II	Bicuspid AV and mild to moderate AVS	44	Progression of AV calcium at 6 months

CKD, cronic kidney disease; CAC, Coronary Artery Calcification; AVC, aortic valve calcification; AVS, aortic valve stenosis.
